# F92. COMPARISONS BETWEEN CANNABIS USERS AND NON-USERS PATIENTS WITH FIRST-EPISODE PSYCHOSIS IN NEUROCOGNITIVE FUNCTIONING: A META-ANALYSIS

**DOI:** 10.1093/schbul/sby017.623

**Published:** 2018-04-01

**Authors:** Teresa Sanchez-Gutierrez, Belén Fernandez-Castilla, Sara Barbeito, Juan Antonio Becerra, Ana Calvo

**Affiliations:** 1 Universidad Internacioal de La Rioja (UNIR); 2 KU Leuven, University of Leuven

## Abstract

**Background:**

Patients with first episode psychosis (FEP) frequently report cannabis use although its effects on cognitive functioning are still unclear. Several studies suggest a decrease in the executive function, verbal memory and working memory of FEP cannabis users (González-Pinto et al., 2016; Mata et al. 2008) while other studies show improvements in the neurocognitive function of this group (Setién-Suero et al., 2017, Cuhna et al., 2013, Leeson et al., 2012, Yücel et al., 2012; Rodríguez-Sánchez et al., 2010) or even absence of neurocognitive differences between FEP cannabis users and non-users (Burgra et al., 2013). This meta-analysis aims to explore the magnitude of effect of cannabis use on neurocognition in patients with FEP.

**Methods:**

Articles for consideration were identified through extensive literature searches using online databases, which included PubMed, Medline and PsychInfo. The search was limited to English language articles. The used keywords were: “first episode psychosis” OR, “neurocognition and cannabis”, in combination with a number of neuropsychology-related terms including “neurocog*” and “neuropsycholog*”. Given that other substances including alcohol, cocaine, and stimulants are associated with altered cognitive performance, studies in which participants met for poly-substance use disorders, even if there was preferential use towards cannabis, were excluded. Eight studies from 2008 to 2017 met inclusion criteria from a total sample of 16 initial studies. Five hundred and eighteen of these participants were cannabis users with FEP, and 639 were patients with no cannabis use. A total of 58 effect sizes of neuropsychological test variables were categorized into 4 cognitive domains (premorbid IQ, executive functioning, working memory and verbal memory and learning). Age of first cannabis use, duration of cannabis use, percentage of males and age were abstracted and assembled as moderator variables. Standardized mean differences were computed for each cognitive domain between cannabis-using patients and patients with no history of cannabis use. Negative effect sizes would display better cognitive functioning of non-cannabis users. We employed a meta-analytic three level model to combine effect sizes across studies.

**Results:**

Effect sizes were not significantly different from zero in any of the neurocognitive domains when FEP cannabis users and non-users patients were compared [working memory (d= -0.03, SE=0.15, CI = -0.33–0.26, p=0.83), executive function (d= 0,14, SE=0.16, CI = -0.17–0.45, p=0.37), verbal memory and learning (d= 0.04, SE=0.15, CI = -0.25–0.33, p=0.27) and premorbid IQ (d= 0.06, SE=0.09, CI = -0.24–0.12, p=0.50)]. Only one moderator variable resulted significant in the executive function denoting superior performance in FEP cannabis-using patients as they were older.

**Discussion:**

Cannabis use is not related to an ameliorated or improved neurocognitive functioning in patients with a first episode psychosis. This is consistent with previous studies which showed absence of differences in the neurocognitive functioning between FEP cannabis users and nonusers (Burgra et al., 2013). However, it has been demonstrated that continued cannabis intake worsens cognitive performance although some of the FEP patients had better premorbid capacities (González-Pinto, 2016). Moreover, the doses and the different types of cannabis preparations may interfere the present results. Meta-analysis on longitudinal studies which include these potential moderator variables may be performed in the future.

